# All paths lead to hubs in the spectroscopic networks of water isotopologues H_2_^16^O and H_2_^18^O

**DOI:** 10.1038/s42004-024-01103-8

**Published:** 2024-02-16

**Authors:** Roland Tóbiás, Meissa L. Diouf, Frank M. J. Cozijn, Wim Ubachs, Attila G. Császár

**Affiliations:** 1 Laboratory of Molecular Structure and Dynamics, Institute of Chemistry, ELTE Eötvös Loránd University and HUN-REN-ELTE Complex Chemical Systems Research Group, H-1117 Budapest, Pázmány Péter sétány 1/A, Hungary; 2 Department of Physics and Astronomy, LaserLaB, Vrije Universiteit, De Boelelaan 1081, 1081 HV Amsterdam, The Netherlands

**Keywords:** Physical chemistry, Theoretical chemistry

## Abstract

Network theory has fundamentally transformed our comprehension of complex systems, catalyzing significant advances across various domains of science and technology. In spectroscopic networks, hubs are the quantum states involved in the largest number of transitions. Here, utilizing network paths probed via precision metrology, absolute energies have been deduced, with at least 10-digit accuracy, for almost 200 hubs in the experimental spectroscopic networks of H_2_^16^O and H_2_^18^O. These hubs, lying on the ground vibrational states of both species and the bending fundamental of H_2_^16^O, are involved in tens of thousands of observed transitions. Relying on the same hubs and other states, benchmark-quality line lists have been assembled, which supersede and improve, by three orders of magnitude, the accuracy of the massive amount of data reported in hundreds of papers dealing with Doppler-limited spectroscopy. Due to the omnipresence of water, these ultraprecise line lists could be applied to calibrate high-resolution spectra and serve ongoing and upcoming space missions.

## Abstract (web summary)

Ultraprecise spectral line lists of molecules such as water, omnipresent in various environments, can be used to calibrate high-resolution spectra, which is particularly relevant for exoplanet transit observations. Here, the authors use measured spectra and network theory to achieve ultraprecise characterization of H_2_^16^O and H_2_^18^O hubs, the most relevant quantum states within a spectroscopic network.

## Introduction

Network theory^1^, a branch of discrete mathematics useful to investigate large natural and artificial systems, has experienced rapid development and widespread applications over the last two decades^2,3^. In fact, network-based analysis of complex systems, including social communities^4^, ecological associations^5^, cancer genomes^6^, brain connectomes^7^, as well as the COVID-19 pandemic^8,9^, has been a hot topic in contemporary interdisciplinary research. Common to these complex systems is that their representation with a network brings considerable advantages during the exploration of their intrinsic properties. In these large-scale networks, the degrees of the nodes often display an inverse-power-law-like (i.e., heavy-tailed or nearly scale-free^2,10,11^) distribution, indicating the presence of a limited number of unusually high-degree nodes, called hubs (note that the degree of a node is the number of edges attached to it).

More than a decade and a half ago, network theory found its way into high-resolution molecular spectroscopy, as well^12,13^. In a spectroscopic network (SN), the nodes are quantum states and the edges are lines/transitions among these states (unless otherwise noted, the words “line” and “transition” refer here to a one-photon, electric-dipole-allowed rovibrational line, though SNs may also contain other types of transitions). According to the Ritz principle^14^, a transition frequency is proportional to the energy difference of the upper and lower states, allowing the determination of empirical energies from a set of measured line positions.

Due to their outstanding significance in numerous scientific and engineering applications (e.g., astronomy, atmospheric sensing, radiative transfer, and combustion chemistry), information related to experimental and/or computed transitions are collated into large-scale, line-by-line spectroscopic datasets, such as those forming the basis of the HITRAN database^15^. As shown repeatedly^13,16–21^, network theory offers a powerful instrument for the maintenance of these databanks, with the unique ability to analyze the self-consistency of the transitions deposited^16,18,19,21^.

Experimental SNs, that is networks built upon measured transitions, possess a heavy-tailed degree distribution^13,16^. This does not hold for SNs formed by complete theoretical line lists, but once lines below an experimentally reasonable detection limit are excluded, their degree distribution becomes heavy-tailed. Since hubs are typically the lower states in a vast number of lines, the accurate knowledge of their energies is of paramount importance when deriving accurate upper-state energies, for example, through the use of the combination-difference approach popular among spectroscopists.

When pursuing high accuracy, modern precision-spectroscopy techniques^22–32^, which often rely on frequency combs and cavity enhancement under non-linear (Doppler-free) absorption conditions, are extremely valuable as they allow to lower the frequency uncertainties down to the kHz level. This high accuracy is helpful in a wide range of applications, like frequency metrology^26,31^, assessing the time stability of physical constants^33^, searching for new physics beyond the standard model^34^, and analyzing velocity fields in star-forming regions^35^.

In this article, the spectral hubs of H_2_^16^O and H_2_^18^O, the two most abundant water isotopologues, are scrutinized via spectroscopic-network-assisted precision spectroscopy (SNAPS)^36^. For these astronomically and atmospherically relevant triatomic molecules, the energies of the hubs are ascertained from well-designed network paths (i.e., sequences of connected, unrepeated transitions and states). These paths involve Lamb-dip lines yielded by this study or collected from the literature^36–41^. The set of transitions targeted for measurement is composed via an inventive design scheme and the Lamb dips are detected with two^26,32^ noise-immune cavity-enhanced optical heterodyne molecular spectroscopy (NICE-OHMS)^22,23^ setups equipped with three probe lasers.

## Results and discussion

### Identification of hubs in the H_2_^16^O and H_2_^18^O networks

Let us first define what a spectral hub is, as there is no strict definition for hubs in network theory^1^. Our definition followed in this study is that a hub is a state with degree *d *≥ d^*^, where d^*^ is the largest integer for which at least 1 % of the states have at least d^*^ edges.

**Fig. 1:**
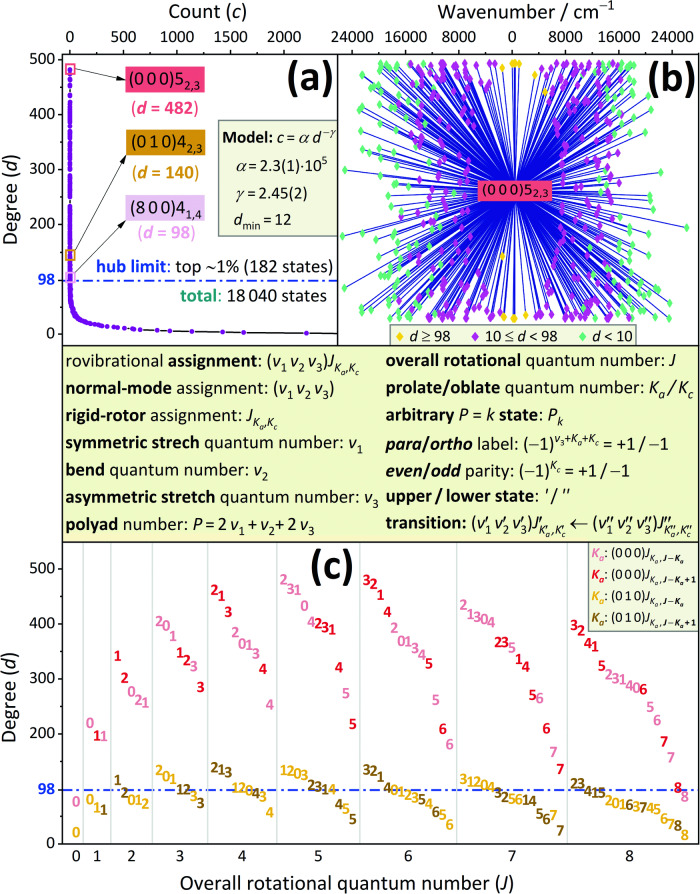
Demonstration of the degree distribution in the experimental spectroscopic network of H_2_^16^O. The central panel specifies the most important terms and symbols used throughout this study. Panel **a** shows a typical discrete, heavy-tailed degree distribution. This distribution pertains to a subnetwork of the H_2_^16^O network reported in ref. ^42^, where only the pure experimental lines are included, contracting each multiple edge into a single one (for the concrete subnetwork, see Supplementary Data 1). The highest-degree hubs are given with different colors in panel **a** for the three vibrational bands of the hub list. The degrees *d* follow an inverse power-law-like model down to $d\ge {d}_{\min }$^13,16^, whose parameters (with their last-digit 1*σ* uncertainties in parentheses) are also shown in panel **a**. Panel **b** illustrates the neighboring states of the (0 0 0)5_2,3_ hub, with wavenumbers on the top horizontal axis and incident nodes presented as colored diamonds, with the colors reflecting their degrees. Panel **c** depicts the *J* dependence of the degrees for the *P* = 0 and *P* = 1 polyads up to *J* = 8. In panel **c**, the states are denoted, instead of points, via their (one-digit) *K*_*a*_ values, the full assignments can be read from the right-hand-side legend.

Figure 1 shows the degree distribution in the experimental H_2_^16^O network^42^, where all but one of the 182 hubs correspond to the *P* = 0 and *P* = 1 polyads, that is to the ground vibrational state, (0 0 0), and the bend fundamental, (0 1 0), respectively. The single exception is a highly excited rovibrational state of the *P* = 16 polyad, which appears rather unexpectedly in the list of hubs; its occurrence is due to the large number of transitions reported in ref. ^43^. Of the other 181 hubs, 149 and 32 lie on the *P* = 0 and *P* = 1 polyads, respectively. As to the experimental SN of H_2_^18^O^42^, it is characterized by a degree distribution similar to that of Fig. 1a (see Supplementary Data 1). The 6755 states linked by purely experimental lines define 68 hubs, all within the *P* = 0 polyad.

An important goal of this study is to determine highly accurate energies for (a) all the H_2_^18^O hubs and (b) a large number of H_2_^16^O hubs, including all the *P* = 1 hubs. Achieving these objectives strongly relies on recent instrumental developments in our precision-spectroscopy apparatus^32^ and an innovative network-based design scheme described in the following subsection.

### Network-guided precision measurements

**Fig. 2:**
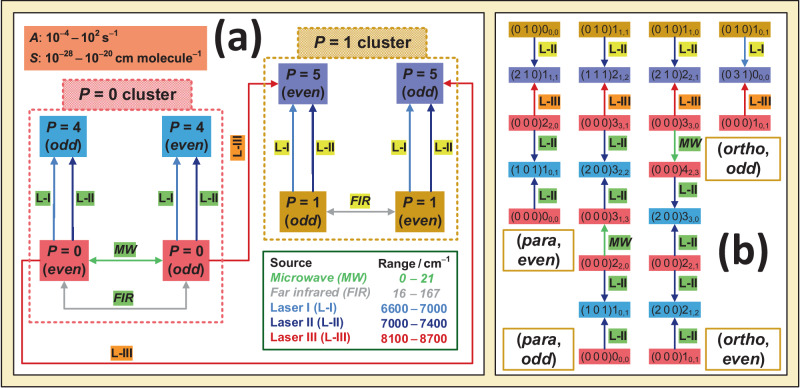
Design scheme used for the precise characterization of hubs in the H_2_^16^O and H_2_^18^O networks. For the general terms and spectroscopic symbols employed, see the central panel of Fig. 1. The colors of the transitions denoted with arrows are shown in a green-framed box collecting the wavenumber ranges of the probe lasers. The apricot box exhibits the Einstein-*A* coefficient and the room-temperature intensity windows accessible in the intervals of the L-I, L-II, and L-III lasers. The highly accurate green and the less accurate gray transitions are taken from refs. ^37–39,53,54^, whereas the light blue, dark blue, and brick-red lines were targeted during this study and in refs. ^36,40^, and ^41^. Panel **a** shows the network-based design scheme, whereby the *even/odd*-parity states of the *P* = 0, *P* = 1, *P* = 4, and *P* = 5 polyads are represented with red, brown, cerulean blue, and lavender blue boxes, respectively. Panel **b** gives examples for paths securing uninterrupted connections from the $(0\,1\,0){[J\le 1]}_{{K}_{a},{K}_{c}}$ states to the *para* and *ortho* rovibrational ground states, (0 0 0)0_0,0_ and (0 0 0)1_0,1_, respectively.

Figure 2 demonstrates an elaborate network-based design scheme constructed to provide ultraprecise energies for the hubs of H_2_^16^O and H_2_^18^O, making use of the three laser ranges accessible in our two NICE-OHMS setups. Figure 2a displays two highly-connected clusters, distinguished by their lower states belonging either to the *P* = 0 or *P* = 1 polyad. For one-photon, dipole-allowed ${P}_{4/5}^{{\prime} }\leftarrow {P}_{0/1}^{{\prime\prime} }$ transitions, such as those measured here, both the *P* = 0 and the *P* = 1 clusters are disconnected subnetworks. To join the even-parity *P* = 0 and *P* = 1 states with their odd-parity counterparts, a few pure rotational ${P}_{0}^{{\prime} }\leftarrow {P}_{0}^{{\prime\prime} }$ and ${P}_{1}^{{\prime} }\leftarrow {P}_{1}^{{\prime\prime} }$ lines should be included in both clusters. A way to make connections between the *P* = 0 and *P* = 1 states is to concatenate the two clusters with Λ pairs of ${P}_{5}^{{\prime} }\leftarrow {P}_{0}^{{\prime\prime} }$ transitions lying within the range of Laser III (a Λ-pair is formed by two lines sharing the same upper state). Connectivity inside the *ortho* and *para* subnetworks could be secured without reliance on less accurate (~30–300 kHz), far-infrared ${P}_{0}^{{\prime} }\leftarrow {P}_{0}^{{\prime\prime} }$ and ${P}_{1}^{{\prime} }\leftarrow {P}_{1}^{{\prime\prime} }$ transitions, introducing a handful of ${P}_{5}^{{\prime} }\leftarrow {P}_{0}^{{\prime\prime} }$ lines into the network.

Due to the absence of observed *ortho* ↔ *para* lines^44,45^, the *ortho* and *para* subnetworks are two separate components (i.e., maximal connected collections of states), preventing the overall experimental connection of the Fig. 2a network. Thus, only relative *ortho* energies can be obtained from the experiments, where the relative energy of an *ortho*/*para* state is specified as the difference between the absolute energy of this state and that of the lowest-energy *ortho*/*para* state. For the empirical derivation of absolute *ortho* energies, one could (i) employ accurate theoretical *ortho*–*para* splittings^36,40^ by creating artificial links between the two components or (ii) add the lowest *ortho* energy of an effective Hamiltonian model to the relative values. In what follows, option (ii) is applied.

To explicate and contextualize Fig. 2a, paths involving the four lowest-lying *P* = 1 states are depicted in Fig. 2b. The top two lines of the four paths are Λ pairs and these pairs introduce links between the sets of *P* = 0 and *P* = 1 states. The bottom parts of these paths are also useful, as they illustrate how the (*ortho*/*para*, *even*/*odd*) states of the *P* = 0 polyad can be attained via microwave and near-infrared transitions from the *ortho*/*para* ground state. For all the *P* = 1 states considered for H_2_^16^O, only ${P}_{5}^{{\prime} }\leftarrow {P}_{0}^{{\prime\prime} }$ and ${P}_{5}^{{\prime} }\leftarrow {P}_{1}^{{\prime\prime} }$ transitions had to be measured during this study, because these states could be connected to those *P* = 0 states already accessible from the *ortho*/*para* ground state through the ultraprecise paths of refs. ^36^ and ^41^. In the case of the H_2_^18^O hubs, the accurate paths formed in ref. ^40^ could also be exploited to decrease the number of target lines.

**Fig. 3:**
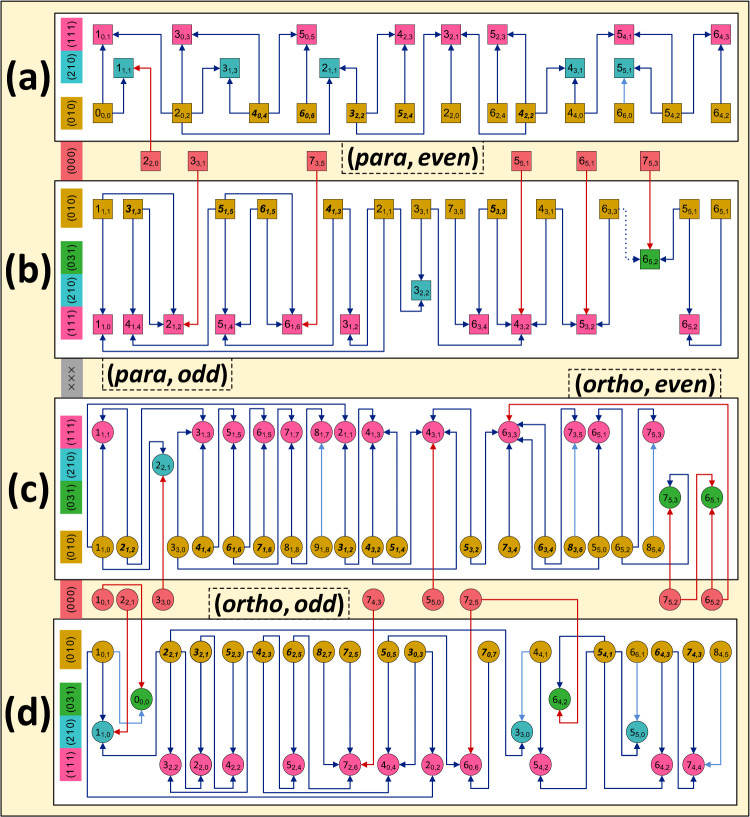
Pictorial overview of the precision lines detected during this study for the H_2_^16^O isotopologue. For the general notions and symbols employed, consult the central panel of Fig. 1. In this actual figure, the *ortho* and *para* states are marked with circles and squares, respectively. For all these states, the ${J}_{{K}_{a},{K}_{c}}$ assignments are written out explicitly, whereas the (*v*_1,_ *v*_2_, *v*_3_) triplets are given in the left-side color legend. Those states with rotational labels typeset in bold italics are hubs within the degree distribution of Fig. 1a. The arrows, whose colors are specified in the green box of Fig. 2a, mean transitions measured during this study with an accuracy of 1.9–38.8 kHz. To the best of our knowledge, the dotted (0 3 1)6_5,2_ ← (0 1 0)6_3,3_ line, see panel **b**, is the least intense (*S* = 4.9 × 10^−28^ cm molecule^−1^) transition that has ever been identified for water isotopologues in saturation. Drawing on the *even*/*odd* and *ortho*/*para* labels of the lower states, the blue lines can be divided into four internally connected subnetworks (see panels **a**–**d**). These subnetworks are linked to some *P* = 0 states through the brick-red lines. Albeit not indicated, these *P* = 0 states are joined via ultraprecise paths, shown in Figs. 3 and 4 of ref. ^41^, to the *ortho*/*para* ground state.

Utilizing our NICE-OHMS setups and the list of targeted transitions, 135/7 absorption lines were observed under saturation for H_2_^16^O/H_2_^18^O (see also Fig. 3 and Table 1). These Lamb dips are characterized by diverse linewidths (half-width at half maximum) of 150–1500 kHz, when measured at room temperature (296 K), a total sample pressure of 0.05–0.55 Pa, and an intracavity power of 1–100 W. The collection of new transitions includes 8, 117, and 17 lines in the regions of Lasers I, II, and III, respectively (see Fig. 2). For all these transitions, line-by-line uncertainties were ascertained. In the case of five H_2_^16^O lines, the pressure shifts were studied explicitly, with the result that all slopes fall within ± 20 kHz Pa^−1^, the highest value found earlier^36,40,41^. The experimental line positions, along with their calculated 1*σ* uncertainties, are deposited in Supplementary Data 1.

**Table 1:** Ultrahigh-accuracy results obtained during this study for the H_2_^18^O isotopologue^*a*^.

Transition (NICE-OHMS)			Lower state (SNAPS)	
Pair	Assignment	Wavenumber/cm^−1^	Relative energy/cm^−1^	Hub?
1 & 2	(2 0 0)8_3,6_ ← (0 0 0)8_2,7_	**7267.323 965 992(67)**	858.159 130 38(19)	yes
	(2 0 0)8_3,6_ ← (0 0 0)9_0,9_	**7232.980 364 473(83)**	**892.502 731 90(21)**	yes
3 & 4	(1 0 1)8_3,5_ ← (0 0 0)8_3,6_	**7237.244 485 887(67)**	977.950 777 34(28)	yes
	(1 0 1)8_3,5_ ← (0 0 0)9_1,8_	**7164.187 247 276(83)**	**1051.008 015 95(30)**	yes
5 & 6	(0 0 2)8_3,6_ ← (0 0 0)8_4,5_	**7270.314 091 144(87)**	1092.881 155 49(24)	yes
	(0 0 2)8_3,6_ ← (0 0 0)9_2,7_	**7188.750 644 337(83)**	**1174.444 602 30(27)**	yes
7 & 8	(2 0 0)9_5,4_ ← (0 0 0)8_6,3_	7197.666 239 152(77)	1375.672 914 85(35)	no
	(2 0 0)9_5,4_ ← (0 0 0)9_4,5_	**7241.894 981 064(87)**	**1331.444 172 94(36)**	yes

^*a*^ Columns 1–3 contain the transitions probed with our NICE-OHMS spectrometers to produce new SNAPS-based energies for four H_2_^18^O hubs. Column 4 comprises the relative energies of the lower states derived via the SNAPS method. Column 5 lists which of the lower states are hubs in the experimental H_2_^18^O network (see Supplementary Data 1). All numerical data typeset in boldface are results of the present study, whereas the plain values are taken from ref. ^40^. The transition wavenumbers and the relative energies are associated with the 1*σ* uncertainties of their last digits in parentheses. The lines of this table form Λ pairs, whose wavenumber differences yield energy differences for their lower states. For each Λ pair, the boldfaced relative energy can be reproduced by forming the sum of the associated wavenumber difference and the other (plain) relative energy. The uncertainties of the relative energies are combined 1*σ* uncertainties (i.e., the square roots of the sum-of-squared uncertainties).

### Ultrahigh-accuracy absolute rovibrational energies

From the dataset of newly determined Lamb-dip lines and those high-accuracy transitions reported in refs. ^36–41^ and ^46–48^, two ultraprecise SNs were built. The H_2_^16^O/H_2_^18^O SNs are formed by 397/208 lines and 317/195 states, forming two (principal) components based on nuclear-spin statistics. Setting the squared line uncertainties as edge weights, a shortest-path search^1^ was carried out from the *ortho*/*para* ground state to the other *ortho*/*para* states. The resulting paths are named here best paths, as they produce the best relative energies and associated uncertainties for their terminal states.

**Fig. 4:**
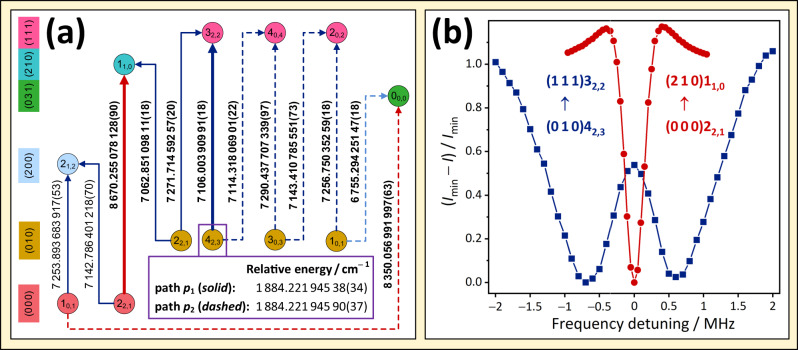
Illustration of the SNAPS procedure in operation for the highest-degree ***P*** **=** **1** hub of H_2_^16^O. The general terms and symbols utilized are defined in the central panel of Fig. 1. Panel **a** contains two disjoint paths from (0 0 0)1_0,1_ to (0 1 0)4_2,3_, *p*_1_ and *p*_2_, where the lines are denoted with solid and dashed arrows, respectively. For the transitions and the states of panel **a**, the same formalism is adopted as in Fig. 3. The numbers on the arrows are wavenumbers in cm^−1^, with the 1*σ* uncertainties of the last digits in parentheses. The boldfaced values were observed during this study, whereas the other two wavenumbers are taken from ref. ^36^. Relying on *p*_1_ and *p*_2_, two independent estimates can be derived for the (0 1 0)4_2,3_ relative energy, which are presented in the violet box of panel **a**. In fact, *p*_1_ and *p*_2_ form a 12-membered cycle with a discrepancy of 5.1(50) × 10^−7^ cm^−1^ [15.4(151) kHz]. For technical details on the determination of relative energies and discrepancies, Eqs. (1–5). Panel **b** presents two typical Lamb dips, a regular and an inverted profile, recorded with our NICE-OHMS apparatus. To aid their comparison, both spectra are mapped onto the $({I}_{\min }-I)/{I}_{\min }$ intensity scale, where *I* means the intensity at a detuning point for a particular line, and ${I}_{\min }$ is the lowest intensity in the ± 2 MHz detuning region. As documented in ref. ^41^, inverted profiles occur for transitions with large (>0.1 s^−1^) Einstein-*A* coefficients.

Figure 4 illustrates two disjoint paths, *p*_1_ and *p*_2_, for the highest-degree *P* = 1 hub of H_2_^16^O, together with the Lamb-dip spectra of two typical lines on *p*_1_. These two paths specify a cycle (that is a series of connected lines and states, where each state has exactly two neighboring states), whose discrepancy satisfies Eq. (5) of the Methods section, corroborating the high accuracy of the underlying transitions. The two paths provide basically the same relative energies, of which the lower-uncertainty datum is adopted as the final estimate. It is worth emphasizing that the average uncertainty of the relative energies is around 3.5 × 10^−7^ cm^−1^ in the ultraprecise H_2_^16^O/H_2_^18^O SNs, implying a 10–1000 times improvement over previous, Doppler-limited measurement results.

To switch to absolute energies, the relative *ortho* energies need to be shifted with the lowest *ortho*-H_2_^16^O and *ortho*-H_2_^18^O energy, determined to be 23.794 361 22(25)^36^ and 23.754 904 61(19) cm^−1^ ^40^, respectively. After this conversion, accurate absolute energies could be derived for 55/100 % of the hubs in the *P* = 0 polyad of H_2_^16^O/H_2_^18^O, as well as for all the *P* = 1 hubs of H_2_^16^O. Thus, 115/68 of the H_2_^16^O/H_2_^18^O hubs are now known with an accuracy of a few times 10^−7^ cm^−1^, which are incident to 31 204/14 637 unique transitions inside the experimental H_2_^16^O/H_2_^18^O networks (see Supplementary Data 1). Furthermore, the new measurements appended altogether 61 states of the *P* = 1 polyad to the ultraprecise H_2_^16^O network, representing a complete set up to *J* = 6 and delivering an improved estimate, 1 594.746 272 93(25) cm^−1^, for the bending fundamental of H_2_^16^O. For both H_2_^16^O and H_2_^18^O, the best paths, together with the ultrahigh-accuracy energies and their uncertainties, are available in Supplementary Data 1.

### Ultraprecise reference line lists

Exploiting the ultraprecise paths composed during this study, the benchmark-quality predicted line lists of H_2_^16^O^41^ and H_2_^18^O^40^ have been enlarged significantly. In the updated lists, a transition is represented with a differential path, which can be generated from the disjoint lines of the two best paths visiting the upper and lower states. These differential paths yield the transition wavenumbers and kHz-level uncertainties for the predicted lines. The updated line lists of H_2_^16^O/H_2_^18^O, placed in Supplementary Data 1, include 4742/1690 entries, out of which 1907/878 lines have relatively high intensity, *S* ≥ 10^−25^ cm molecule^−1^. These ultraprecise databases span the 0–3868/0–1249 and 4430–12666/5909–8379 cm^−1^ wavenumber ranges, and have 2328/822 and 2414/868 lines, respectively.

The high-intensity transitions of the lower wavenumber interval represent ${P}_{0}^{{\prime} }\leftarrow {P}_{0}^{{\prime\prime} }$, ${P}_{1}^{{\prime} }\leftarrow {P}_{0}^{{\prime\prime} }$, and ${P}_{1}^{{\prime} }\leftarrow {P}_{1}^{{\prime\prime} }$ lines, of which the latter two stem from our newly measured H_2_^16^O transitions. A great number of these ${P}_{0/1}^{{\prime} }\leftarrow {P}_{0/1}^{{\prime\prime} }$ lines establish links between pairs of hubs (see, e.g., the connections of the middle state with a few yellow states in Fig. 1b). Similar to social networks^10^, the hubs of the experimental H_2_^16^O and H_2_^18^O SNs are also highly interconnected, due to preferential attachment driven by quantum-mechanical selection rules^16^.

While our line catalogs contain only one-photon transitions allowed by dipole and nuclear-spin selection rules, other types of extremely weak lines, like two-photon, quadrupole, and *ortho*-*para* transitions, could also be deduced with 10–20 kHz uncertainties from the accurate absolute energies. It must be stressed that measuring quadrupole and/or two-photon ${P}_{4/5}^{{\prime} }\leftarrow {P}_{0/1}^{{\prime\prime} }$ lines based on these accurate predictions would be truly advantageous, as they could ensure stronger connections between the loosely attached *even*- and *odd*-parity *P*_0/1_ states.

## Concluding remarks

An important message of this study is that combining network theory with precision spectroscopy provides an outstanding tool for the ultraprecise characterization of hubs, the most relevant quantum states in an experimental spectroscopic network. In fact, utilization of a sophisticated design scheme and the detection of close to 150 new Lamb dips for H_2_^16^O and H_2_^18^O resulted in absolute energies with a typical relative accuracy of 10^−10^ for the complete set of H_2_^18^O hubs, as well as for all the H_2_^16^O hubs on the bending fundamental and a large part of the hubs within its ground vibrational state. To access the remaining H_2_^16^O hubs with ultrahigh accuracy, further observations are needed, for which some target lines might fall outside the ranges of the three lasers of this study.

Owing to the efficiency of the network approach, the benchmark-quality predicted line lists of H_2_^16^O/H_2_^18^O include 4.8/4.2 times more transitions with high (*S* ≥ 10^−25^ cm molecule^−1^) intensities than the set of direct Lamb-dip measurements. These large multiplication factors justify our efforts to achieve overall connectivity within the *ortho*/*para* subnetworks. The new benchmark line lists of the two water isotopologues encompass a considerable number of frequency standards, suitable for the calibration of Doppler-broadened spectra (note that our previous H_2_^16^O line list^36^ has already been invoked in ref. ^49^ to report corrected Fourier-transform line positions). Furthermore, those transitions falling into the mid-infrared bandwidth of the James Webb Space Telescope, 5–28.5 μm (350–2000 cm^−1^), would be optimal targets for tracing water isotopologues in exoplanetary atmospheres and improve our understanding of masers within the bending fundamental^50,51^.

## Methods

### Theory

The SNAPS approach^36^ employed in this study proved to be ideal to maximize the utility of results extracted from precision-spectroscopy measurements^40,41^, where the so-called primary line parameters (wavenumbers, intensities, and Einstein-*A* coefficients) are restricted to narrow ranges. The SNAPS procedure consists of the following steps: (a) construction of paths and cycles from accurate literature transitions, augmented with target lines in specific intervals of the primary line parameters, (b) detection of the target transitions, and (c) analysis of the paths and cycles comprising the old and new lines. For the selection of the targeted H_2_^16^O and H_2_^18^O lines, the primary line parameters were taken from ref. ^42^. To avoid extreme scanning times, refined initial wavenumbers coming from Λ-correction schemes^40^ were provided for transitions with *S* < 10^−25^ cm molecule^−1^.

During step (c), an accurate energy difference is calculated for each path between its starting and ending states by using the Ritz principle in a sequential fashion:

1 $${E}_{{j}_{L+1}}-{E}_{{j}_{1}}=\mathop{\sum }\limits_{l=1}^{L}{s}_{l}{\tilde{\nu }}_{{i}_{l}},$$

which is characterized with a well-defined 1*σ* uncertainty,

2 $$u({E}_{{j}_{L+1}}-{E}_{{j}_{1}})=\sqrt{\mathop{\sum }\limits_{k=1}^{L}{u}^{2}({\tilde{\nu }}_{{i}_{k}})}.$$

In these formulas, (i) *L* designates the path length, (ii) *i*_1_, *i*_2_,..., *i*_*L*_ and *j*_1_, *j*_2_,..., *j*_*L*+1_ correspond to the indices of transitions and states occurring on this path, respectively, (iii) *E*_*n*_ indicates the energy of the *n*th state, (iv) ${\tilde{\nu }}_{m}$ and $u({\tilde{\nu }}_{m})$ are the wavenumber of the *m*th transition and its 1*σ* uncertainty, respectively, and (v) ${s}_{{i}_{k}}$ is the sign pertaining to the *i*_*k*_th line of this path. The ${s}_{{i}_{k}}$ signs are chosen such that the energies of the intermediate states, ${E}_{{j}_{k}}$ with 1 < *k* ≤ *L*, will not appear in Eq. (1). When the starting state of this path coincides with the lowest-energy state of a particular nuclear-spin isomer, then the predicted energy difference is identical to the relative energy of the ending state.

If the path behind Eqs. (1) and (2) is extended with an extra transition, indexed with *i*_*L*+1_, a cycle is obtained. In this case, there are two predictions for ${E}_{{j}_{L+1}}-{E}_{{j}_{1}}$, and their unsigned difference is the discrepancy of the underlying cycle,

3 $$D=\left\vert \mathop{\sum }\limits_{k=1}^{L+1}{s}_{k}{\tilde{\nu }}_{{i}_{k}}\right\vert ,$$

associated with a definitive 1*σ* uncertainty,

4 $$u(D)=\sqrt{\mathop{\sum }\limits_{k=1}^{L+1}{u}^{2}({\tilde{\nu }}_{{i}_{k}})}.$$

To decide whether the discrepancy is within an acceptable limit, a Student-*t* test can be performed, which prescribes

5 $$D\le {t}_{{{{{{{{\rm{crit}}}}}}}}}u(D),$$

whereby *t*_crit_ means the critical Student-*t* factor (*t*_crit_ ≈ 2 for 95 % significance level). When a cycle does not satisfy Eq. (5), there is a conflict among the transitions of this cycle (note that multiple measurements can be viewed as two-membered trivial cycles). Hence, this compatibility criterion makes cycles useful for the verification of the internal accuracy of ultraprecise SNs.

### Experiment

As to the most important instrumental aspects of this study, the majority of the lines within the range of Laser II were interrogated with a NICE-OHMS setup described in refs. ^26,36^, and ^41^. In this setup, an infrared diode laser is operated at 1.4 μm, combined with a high-finesse cavity. This laser is modulated at 305 MHz to yield side-band signals and at 20 MHz for its lock into the cavity via a Pound–Drever–Hall stabilization scheme. Due to the presence of highly reflective mirrors, of which one is dithered at a low (405 Hz) frequency, the intracavity power can be varied up to 150 W. The spectroscopic signal is demodulated by a lock-in system (Zurich Instruments; HF2LI). An optical frequency comb, disciplined by a cesium atomic clock, is also utilized in this apparatus to stabilize the infrared laser and to reach sub-kHz precision on the frequency scale.

For the rest of the Lamb-dip measurements, another NICE-OHMS spectrometer was deployed, which was actually designed to probe a very weak (*A* = 1.3 × 10^−7^ s^−1^ and *S* = 1.6 × 10^−27^ cm molecule^−1^) quadrupole line of H_2_ under saturation^32^. The main features of the latter setup are as follows: (i) temperature control in the optical cavity, down to the mK level, avoiding background noise caused by residual amplitude modulation in the measured signal, (ii) efficient vibration isolation, ensuring high stability and accuracy during long-term averaging of multiple scans, and (iii) greatly improved sensitivity, characterized by a finesse of 350 000, a maximum intracavity laser power up to 10 kW, a beam waist of 542 μm, and a mirror reflectivity of *R* > 0.999 99. This advanced setup proved to be sensitive enough to yield a minimum uncertainty of 1.9 kHz for the detected lines and record an extremely weak (*A* = 1.7 × 10^−2^ s^−1^ and *S* = 4.9 × 10^−28^ cm molecule^−1^) transition for H_2_^16^O.

The uncertainties of the line positions reported in this study are affected by numerous experimental factors arising mostly from homogeneous broadening processes^52^. To determine the 1*σ* uncertainty of an observed frequency, *δ*, the following uncertainty budget is applied:

6 $$\delta =\sqrt{{\delta }_{{{{{{{{\rm{stat}}}}}}}}}^{2}+{\delta }_{{{{{{{{\rm{day}}}}}}}}}^{2}+{\delta }_{{{{{{{{\rm{pow}}}}}}}}}^{2}+{\delta }_{{{{{{{{\rm{pres}}}}}}}}}^{2}},$$

whereby *δ*_stat_, *δ*_day_, *δ*_pow_, and ${\delta }_{{{{{{{{\rm{pres}}}}}}}}}$ are the statistical, day-to-day, power-shift, and pressure-shift uncertainties, respectively. These individual uncertainty terms are also listed in Supplementary Data 1 for all lines detected during the present study.

### Supplementary information

Description of Additional Supplementary Files

Supplementary Data 1

## Data Availability

The experimental and calculated data of this study are provided as a compressed Supplementary Data 1 file, both for H_2_^16^O and H_2_^18^O. This compressed file contains old and new high-precision lines, high-accuracy predicted line lists, highly-accurate relative energies, basic cycles built upon the new and old high-precision measurements, the connection lists of the experimental spectroscopic networks considered, the degree distributions of the states, and the high-accuracy absolute energies of the hubs. Data behind Figs. 1–4 can also be found in Supplementary Data 1.

## Code availability

The computer codes used for the selection of target lines are available, upon reasonable request, from the second corresponding author (AGC).
