# Supplementary material for: All paths lead to hubs in the spectroscopic networks of water isotopologues H216O and H218O
Source: Commun Chem. 2024 Feb 16;7:34. doi: 10.1038/s42004-024-01103-8 (PMC10873357; doi:10.1038/s42004-024-01103-8)
Supplement: Supplementary file 1 — Description of Additional Supplementary Files [file 42004_2024_1103_MOESM1_ESM.pdf]

Description of Additional Supplementary Files

File name: Supplementary Data 1

Description: This file contains experimental and calculated data and data behind Figs. 1–4.
