# Supplementary material for: All paths lead to hubs in the spectroscopic networks of water isotopologues H216O and H218O
Source: Commun Chem. 2024 Feb 16;7:34. doi: 10.1038/s42004-024-01103-8 (PMC10873357; doi:10.1038/s42004-024-01103-8)
Supplement: Supplementary file 2 — Supplementary Data 1 [file 42004_2024_1103_MOESM2_ESM.zip › SupplementaryData1/H218O/Figure_S1.pdf]

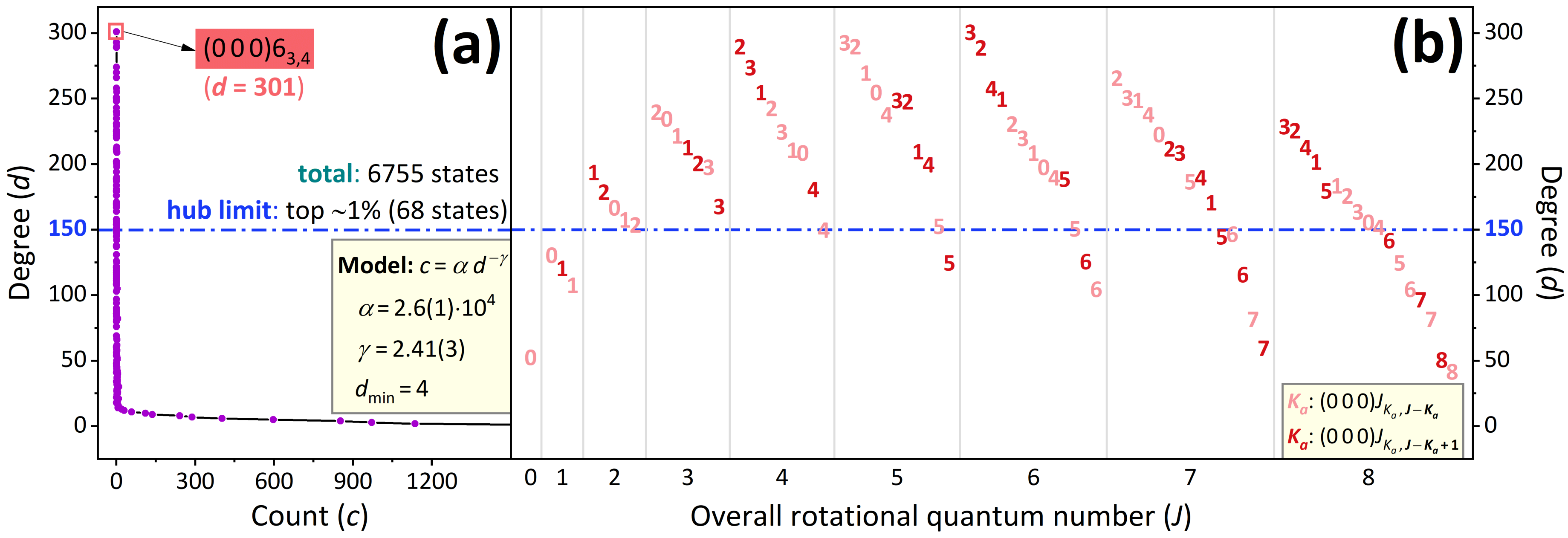


**Figure S1: Demonstration of the degree distribution in the experimental spectroscopic network of the $H_2{}^{18}O$ isotopologue.** Panel (a) exhibits a discrete, heavy-tailed degree distribution. This distribution pertains to a subnetwork of the spectroscopic network reported in 20FuToTePo, whereby only the purely experimental transitions are included, contracting every multiple edge into a single one. The highest-degree hub is shown in the light red box of panel (a). The degrees follow an inverse power-law-like model down to $d \geq d_{\min}$, whose parameters (with their last-digit uncertainties in parentheses) are also indicated in panel (a). Panel (b) depicts the $J$ dependence of the degrees for the $P = 0$ polyad up to $J = 8$. In panel (b), the states are displayed, instead of points, *via* their (one-digit) $K_a$ values, for which the full assignments can be read from the right-hand-side color legend.
